# Antimicrobial Stewardship Program in Hospitals: Manual of Procedures

**DOI:** 10.1093/jacamr/dlz003

**Published:** 2019-04-08

## Abstract

**Graphical Abstract:**
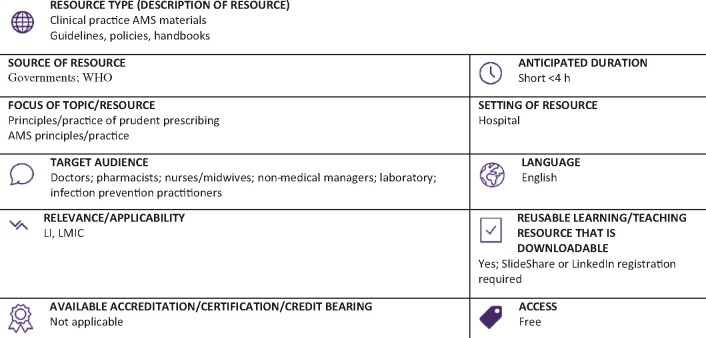

**Resource web link: https://www.slideshare.net/phicna2005/doh-antimicrobial-stewardship-program-in-hospitals-manual-of-procedures-mop-2016** (Full classification scheme available at: http://bsac.org.uk/wp-content/uploads/2019/03/Educational-resource-review-classification-scheme.pdf)

**WHO region and country (World Bank):** Western Pacific, Philippines (LMIC)

## Peer review commentary

This is a very useful guide to the key elements of a hospital-based antimicrobial stewardship (AMS) programme. There is a clear description of the rationale for implementing a stewardship programme, and an explanation of the key elements of such a programme. It is very much based on traditional models of hospital practice, with suggested/recommended types of consults and interventions being paper- or telephone-based. Traditional roles for medics (prescribers or providing consults/reviewing patients), pharmacists (focused on interventions relating to the antimicrobial therapy), not much involvement of nursing staff other than ensuring that the medication that they are administering is approved. Focused on the key interventions of intravenous-to-oral switch, de-escalation, pre-authorization, restriction and hard stop, with useful examples of documentation to support these. Examples of pathways for pneumonia and sepsis. Outline of the key knowledge requirements of staff in the AMS team, as well as the basics required for non-AMS healthcare professionals and public/patients. Examples of audit tools.

Overall, a very useful resource for those who are planning on introducing an AMS programme into their hospital, with advice and guidance on interventions, monitoring, audit and governance. If you have an AMS programme already in your institution, there is unlikely to be anything new here for you.

